# How to Build Transcriptional Network Models of Mammalian Pattern Formation

**DOI:** 10.1371/journal.pone.0002179

**Published:** 2008-05-14

**Authors:** Chrissa Kioussi, Michael K. Gross

**Affiliations:** 1 Department of Pharmaceutical Sciences, College of Pharmacy, Oregon State University, Corvallis, Corvallis, Oregon, United States of America; 2 Department of Biochemistry and Biophysics, College of Science, Oregon State University Corvallis, Corvallis, Oregon, United States of America; University of Nottingham, United Kingdom

## Abstract

**Background:**

Genetic regulatory networks of sequence specific transcription factors underlie pattern formation in multicellular organisms. Deciphering and representing the mammalian networks is a central problem in development, neurobiology, and regenerative medicine. Transcriptional networks specify intermingled embryonic cell populations during pattern formation in the vertebrate neural tube. Each embryonic population gives rise to a distinct type of adult neuron. The homeodomain transcription factor Lbx1 is expressed in five such populations and loss of Lbx1 leads to distinct respecifications in each of the five populations.

**Methodology/Principal Findings:**

We have purified normal and respecified pools of these five populations from embryos bearing one or two copies of the null Lbx1^GFP^ allele, respectively. Microarrays were used to show that expression levels of 8% of all transcription factor genes were altered in the respecified pool. These transcription factor genes constitute 20–30% of the active nodes of the transcriptional network that governs neural tube patterning. Half of the 141 regulated nodes were located in the top 150 clusters of ultraconserved non-coding regions. Generally, Lbx1 repressed genes that have expression patterns outside of the Lbx1-expressing domain and activated genes that have expression patterns inside the Lbx1-expressing domain.

**Conclusions/Significance:**

Constraining epistasis analysis of Lbx1 to only those cells that normally express Lbx1 allowed unprecedented sensitivity in identifying Lbx1 network interactions and allowed the interactions to be assigned to a specific set of cell populations. We call this method ANCEA, or active node constrained epistasis analysis, and think that it will be generally useful in discovering and assigning network interactions to specific populations. We discuss how ANCEA, coupled with population partitioning analysis, can greatly facilitate the systematic dissection of transcriptional networks that underlie mammalian patterning.

## Introduction

The patterning and specification process that generates distinct neuronal cell types in the spinal cord begins as the neural tube is formed from the proliferative neuroepithelium. Signaling centers induce asymmetric expression patterns of sequence specific transcription factors (SSTFs) along the dorsal-ventral axis of the early neural tube. The expression patterns overlap and form discrete boundaries so that eleven progenitor laminae, each of which expresses a distinct combination of SSTFs, can be defined in the ventricular zone. The proliferating cells of the ventricular zone shed postmitotic cells into the marginal layer from embryonic day (E) 9.5 to E13 of mouse development. Each progenitor lamina produces at least one postmitotic cell population. that is defined by a new combinatorial code of SSTF expression. The eleven postmitotic populations that emerge are named dI1-dI6, V0-V3, and M [1]–[8].

Additional mechanisms contribute to the diversification of cell types in the developing neural tube. For example, individual progenitor layers either produce different postmitotic populations at different developmental times, or postmitotic mechanisms produce different SSTF codes, and hence new populations, from single, nascent, postmitotic populations [9]–[15]. Furthermore, differential expression of *Hox* genes along the anterior-posterior (A–P) axis produces different neuronal populations from a given dorsal-ventral (D–V) lamina at different axial levels [16]–[18]. Although the full complement of populations is not completely characterized, it appears they can be represented by SSTF “expression codes”.

At least 66 SSTFs have been invoked in the neural tube patterning process. These include 42 homeodomain, 11 basic helix-loop-helix, and 8 zinc finger SSTFs. Functional perturbations such as gene knock-outs in mice or overexpression in chick embryos have been performed for at least 47 of these SSTFs and many genetic interactions among these SSTFs have been defined. A high degree of recursive linkage between SSTFs in this system appears to exist. However, a population partitioning analysis (PPA) identified 200 additional SSTFs with the same degree of differential expression as the known set and estimated that 500–700 of the 1700 annotated SSTFs in the genome are active nodes in the genetic regulatory network (GRN) of neural tube patterning [19].

Network models are developed to understand the functional organization of complex systems [20]. Specialized software allows complex and evolving datasets, of expression and epistasis information, to be accurately tracked, and aids in decoding the underlying logic of developmental GRNs [21]–[25]. GRNs contain evolutionarily inflexible subcircuits, called kernels, which consist of SSTF nodes with highly recursive linkages and which specify spatial domains in which a body part will form [26]. The SSTF expression codes that are used to spatially define transient neural tube populations are transiently stable in spatial domains during development. Thus, the expressed SSTFs that define a population are predicted to be nodes of a specific network kernel. Transitions between SSTF expression codes, such as those that occur between progenitor laminae and the emergent postmitotic populations, therefore represent transitions between kernels. Removal of one SSTF that participates in a kernel destroys the linkages that stabilize the kernel, and has a catastrophic effect on the development of the respective body part. In line with this model, knocking out SSTFs that contribute to the SSTF codes in the neural tube frequently results in ablation of the respective body part. For example, removing *Isl1* results in the loss of motor neurons [27] and removing *Lbx1* results in the loss of the substantia gelatinosa [9].

Active nodes, in a transcriptional network model of a biological system, represent SSTFs that are differentially expressed in the system. The large number of active SSTF nodes in neural tube patterning suggests that either there are far more kernels than those currently described, or the number of nodes in each kernel is larger than those currently reported in other systems (5–10 SSTFs). We have previously reported how PPA provides a high throughput method to systematically identify active SSTF nodes in a given developmental system [19]. In principle, PPA can be reiterated in progressively constrained sub-systems until no single SSTF is differentially expressed in the sub-system. Such a sub-system is expected to represent a “body part”, specified cell type, network kernel, or stable regulatory state, and can be described by a unique SSTF code.

Active nodes should ultimately be connected by regulatory interactions that reflect the direct interaction of SSTFs with cis-regulatory modules in each stable regulatory state. Developmental cis-regulatory modules in the sea urchin system generally have four to eight diverse inputs [28]. The tools needed to demonstrate direct interactions are currently not amenable to the rapidly changing network states of embryonic mammals and require *a priori* knowledge of all the *cis-* and *trans-*acting components of an interaction. The use of null knock-in alleles allows investigators to compare cells that express a SSTF, in heterozygotes, to the equivalent cells, in mutants, that “should have” expressed the SSTF. Such analyses establish epistatic, rather than direct, interactions between the active nodes in the network model.

Epistatic interactions are useful in organizing and constraining draft network models, which, in turn, can be used to generate specific testable hypotheses about which components are involved in specific, direct regulatory interactions. The rate of discovery of epistatic interactions is currently limited by availability of *in situ* probes and antibodies, as well as by manpower, and will therefore be accelerated by applying high-throughput genomic tools. In this report, we describe how the same genetic tools that are developed for PPA can be employed to define epistatic interactions between active SSTF nodes in a high throughput manner. Acquisition of epistasis data from population pools that are defined by active node expression in embryos assures that only physiologically relevant interactions are recorded into network data sets. In tandem, PPA and active node constrained epistasis analysis (ANCEA) provide a systematic approach to characterize the many kernels created by the patterning mechanism to produce a mammalian body.

## Results

### Flow-Sorting Population Pools by Active Node Constraints

**Figure 1:**
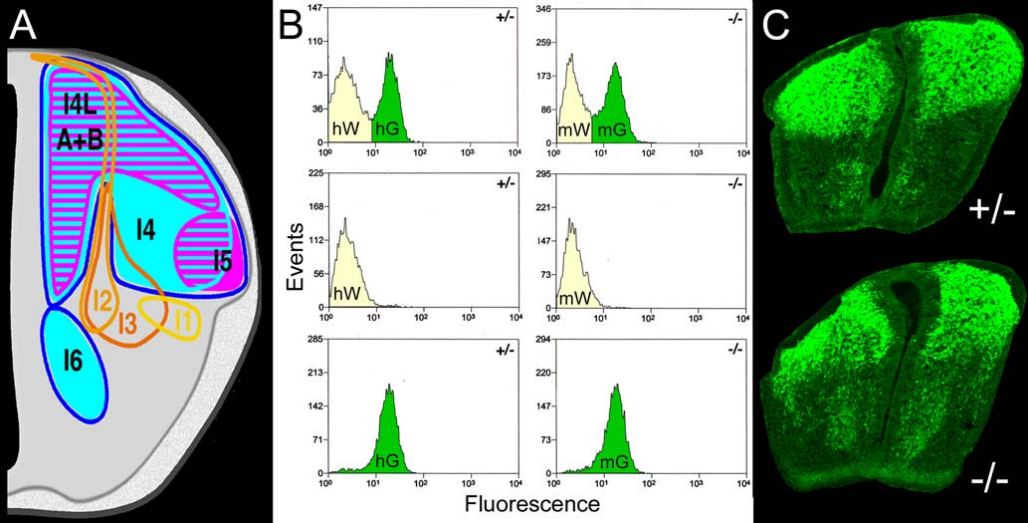
Flow Sorting of Dissociated E12.5 Neural Tubes. (A) Locations of the eight dorsal neural tube populations at E12.5 (right side). The dI1-dI3 populations do not express Lbx1, are born in the most dorsal ventricular zone, and rapidly migrate toward the floor plate (yellow, tangerine, and orange traces). Three small early Lbx1^+^populations, dI4, dI5, and dI6, are born in distinct layers from the middle-dorsal ventricular zone between E10.5 and early E11.5 and move to the regions outlined by magenta and cyan traces by E12.5. Two large late Lbx1^+^populations, dI4L^A^ and dI4L^B^, are born intermingled from the dorsal half of the ventricular zone between late E11.5 and E13 occupy the areas hatched in cyan and magenta. (B) Flow cytometry profiles from neural tubes of heterozygotes (+/−) or mutants (−/−) prior to (top panels) and after (lower panels) sorting. Green or white cell pools are labeled with the four array conditions (hG, hW, mG, mW) they give rise to. (C) Cross sections of heterozygous and mutant neural tubes at the forelimb level at E12.5 stained with GFP antibody.

The *Lbx1* ^GFP^ mouse line [29] provides a robust system for developing genome-wide analyses of epistatic interactions in mammalian embryos. First, the fluorescent cells from E12.5 embryos are abundant and are predominantly from two closely related populations (Fig. 1A). Approximately 80% of Lbx1 expressing neurons in E12.5 neural tubes belong to the two late populations. Second, these two populations have expressed Lbx1, or GFP, for less than 24 hours. Thus, comparisons between mutant and heterozygous sources preferentially reveal immediate molecular consequences of Lbx1 expression, rather than delayed secondary effects. Third, loss of *Lbx1* function leads to known changes in SSTF expression that provide positive controls for the analysis [9], [30].

Fluorescence activated cell sorting (FACS) was used to purify the GFP^+^ (green) and GFP^−^ (white) cells from pools of ten neural tubes of mutant and heterozygote *Lbx1^GFP^* embryos at E12.5 (Fig. 1B). The one hour, serum-free procedure was repeated on eight separate occasions. Four runs showed a high level of reproducibility at the level of timing (Table S1) and RNA quality (data not shown). Green cells constituted 41±6% of the sorted events in both mutants and heterozygotes, supporting the idea that cells are re-specified but not yet apoptotic at this stage. Terminal Transferase dUTP Nick End Labeling (TUNEL) assays have shown that apoptosis in mutants occurs at E13.5 and E14.5 [9]. White cells constituted 53±6% and 52±6% of the sorted events in mutants and heterozygotes, respectively. The ratio of green to white cells accurately reflected the GFP expression observed by histology (Fig. 1C).

Total RNA from three biological replicates of each of the four conditions, heterozygous green (hG), heterozygous white (hW), mutant green (mG) and mutant white (mW), was used to probe Affymetrix Mouse 430 arrays. Data from all twelve arrays were normalized using GC robust multiarray averaging in Genespring software. The analysis focused on probe sets corresponding to SSTFs , which collectively form the key interface between the genetic regulatory information on the DNA and the RNA polymerase II transcriptional machinery and its coregulators [31] and which make up transcriptional network kernels [26]. The annotated SSTF set used in this analysis includes 177 basic, 749 zinc-coordinated, 512 helix-turn-helix, 116 ß-scaffold with minor groove contacts, and 138 other SSTFs and is an updated version of the set described earlier [19]. These 1691 genes were collectively monitored by 3574 probe sets.

### Lbx1 Regulates SSTF Genes in a Cell Autonomous Manner

Region, field, compartment, or cell-type specific selector genes of *Drosophila* are SSTFs. They function cell autonomously in the morphologically distinct, spatial domains of the embryo where they are expressed. Mammalian homologs of these genes are also expressed in spatially constrained ways in embryos. However, the more fluid anatomy of developing mammals generally limits our ability to delineate regions, fields, and compartments on the basis of morphology alone. Consequently, it is usually not possible to determine to what extent the putative mammalian selector genes are functioning cell autonomously.

**Figure 2:**
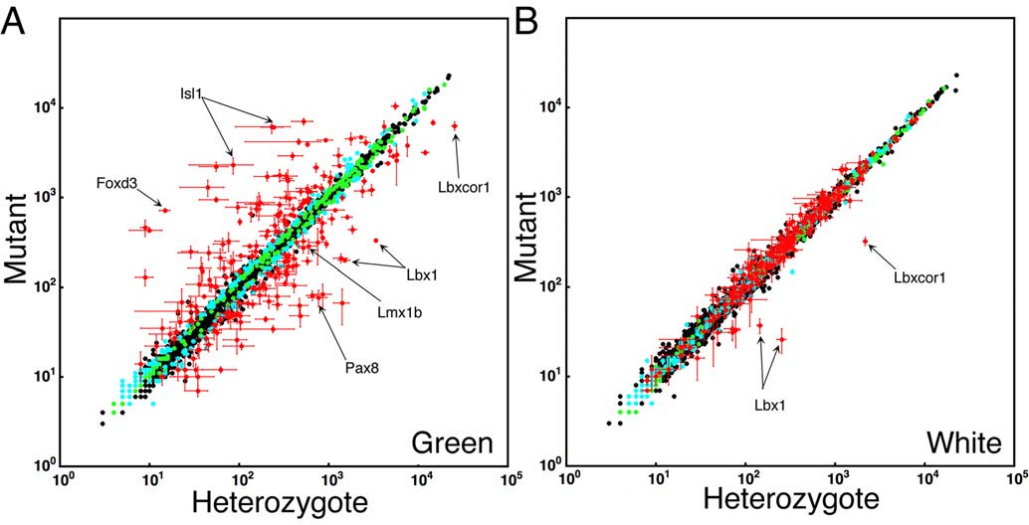
Identification of Lbx1-dependent SSTF Targets. Comparisons were between heterozygotes (x-axis) and mutants (y-axis) in green (left panel) and white (right panel) cell pools. The average intensity values from three independent arrays of three independent isolates are shown. Values for 3574 probe sets corresponding to 1691 SSTF genes are plotted. All 12 arrays were normalized using GCRMA. Color coding, given to each probe set based on their behavior in the green comparison, was maintained in the white comparison. Colored dots represent probe sets that change in a uniform direction in all of the 9 possible 2-way comparisons between the three mutant and three heterozygous arrays of green cells. Probe sets are color coded to show those that passed the 1.3 fold threshold in 35–84 (red), 1–34 (cyan), or zero (green ) permutations of three pair wise, logical AND, comparisons in green cells. Red probe sets were selected to create the tables. Note that red probe sets change in the green comparison but not in the white comparison. The positive controls Lbx1, Lmx1b, Isl1, and Foxd3 are indicated. No probe sets exists for Pax2. However, both Pax8 and Pax5 are regulated in the direction predicted for Pax2.

The average signal intensities of triplicate heterozygous and mutant arrays were compared in scatter plots to reveal *Lbx1*-dependent changes in the expression of SSTF genes (Fig. 2). Heterozygous versus mutant comparisons gave markedly different results in green cells (Fig. 2A), which represent populations that normally express Lbx1, and white cells (Fig. 2B), which represent populations that normally do not express Lbx1. The scatter from the diagonal unity line in the green cell comparison indicates that *Lbx1* regulated many SSTF genes in populations where it is expressed. In contrast, little scatter from the unity line was observed in the white cell comparison, indicating that few SSTF genes are regulated in cells where Lbx1 is not expressed. These results demonstrated that SSTF gene regulation by *Lbx1* in green cell populations have little effect on SSTF gene expression in adjoining white cell populations. Thus, SSTF regulation by *Lbx1* appears largely, or entirely, cell autonomous, consistent with the idea that *Lbx1* functions as a selector gene in a field or compartment defined by its own expression, rather than by morphological boundaries. Any non-cell autonomous regulation is either subtle or occurs between green cell populations. The absence of significant cross-talk between cell populations is consistent with the idea that neural tube pattern formation, during this phase, consists of a series of cell autonomous transitions between stable transcriptional network states.

Only the probe sets for *Lbx1* and its corepressor, *Lbxcor1*, showed significant signal reductions in the white cell comparison, albeit from much lower basal signals than in the green cell comparison. The loss of residual signal from Lbx1 probe sets is expected in null mutants. The concomitant reduction of residual *Lbxcor1* RNA likely reflects a particularly high affinity interaction of Lbx1 with a *cis-*regulatory module of this gene.

### Lbx1 Regulates at Least 6% of SSTF Probe Sets

**Figure 3:**
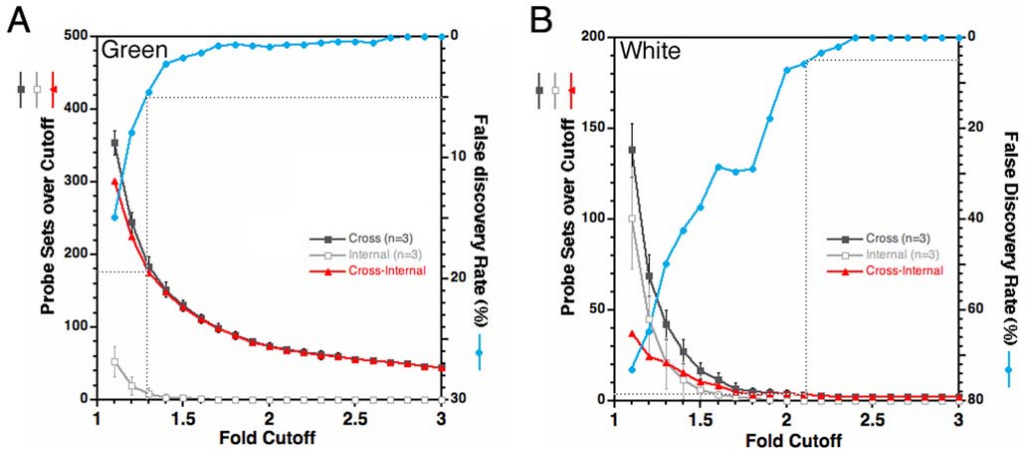
Limiting the Total Target Number by FDR. A) Array data from GFP expressing cells of neural tubes (Green) was compared. These are cells that normally express Lbx1. B) Array data from neural tube cells lacking GFP (White) was compared. These are cells that normally lack Lbx1. In each panel, the results from individual arrays was compared, in a pair-wise manner, between replicate arrays of the same condition (internal), and between arrays of mutant and heterozygote conditions (cross). Triplicate array measurements allowed three pair wise internal-comparisons to be made for each of the four conditions (hG, mG, hW, mW) and nine pair wise cross-comparisons to be made between mutant and heterozygote conditions. The number of probe sets with fold changes at or above a given fold cutoff in three specific internal-comparisons was measured and represents the number of false positives because the comparison was between biological replicates (open squares). The numbers of probe sets with fold changes at or above a given fold cutoff for three specific cross-comparisons between heterozygous and mutant arrays was measured in a comparable manner. Each of the 84 possible permutations of three specific cross-comparisons (of the nine available cross-comparisons) was evaluated at each fold cutoff. The average value obtained from all 84 permutations (filled squares) was plotted in each panel and represents the total number of positives, true plus false. By this method, the number of probe sets above a given fold cutoff was determined in an equivalent manner in cross- and internal-comparisons. The FDR (blue circles) was calculated by dividing the false positives by the total positives. The number of true positives (triangles) was calculated at each fold cutoff by subtracting the false positives from the total positives (see Materials and Methods for a more detailed description of the analysis) .

The large number of SSTF genes that were regulated by *Lbx1* in green cells was surprising in light of the relatively simple genetic diagrams that currently describe neural tube development and the lack of non-cell autonomous regulation. The low level of scatter in the white cell comparison indicated that the noise between biological replicates was very low (Fig. 2B). However, it remained possible that there was simply more variability in gene expression in green samples. The nonparametric permutation fold-scanning method [19] was applied to measure the false discovery rate (FDR) at different fold-cutoffs in green (Fig. 3A) or white (Fig. 3B) cell pools. The details of this method are described under Materials and Methods.

Internal comparisons between triplicate arrays reveal false discoveries arising from combined noise, biological and array processing, of the measurements. The number of false discoveries at each cutoff was very similar in each of the four conditions, as would be expected for noise. Cross comparisons between heterozygous and mutant showed more changes than internal comparisons at each cutoff in both green and white cells. However, in green cells the difference between cross and internal comparisons was far greater than in white cells at all cutoffs and at all FDRs. For example, the FDR reached 5% at the 1.3 fold cutoff in the green comparison. At this cutoff, 180 of the 3108 probe sets (6%) were true positives. In striking contrast, the FDR reached 5% at the 2.1 fold cutoff in the white comparison. At this cutoff, only the *Lbx1* and *Lbxcor1* probe sets were true positives. At the 1.3 fold cutoff in white cells, the FDR was 50% and only 20 SSTFs are true positives. Taken together, these analyses provide a quantitative basis for the assertion that *Lbx1* regulates 6% of SSTF probe sets in Lbx1 expressing populations. The large number of SSTF probe sets with altered expression cannot be dismissed as measurement noise.

### Selecting Interactions for the Network Model

Lists of SSTF genes that are targets of *Lbx1* in green cells (Tables 1, 2) were established by applying similar algorithms as in the permutation fold-scanning analyses above. The database was first queried for probe sets that change in a uniform direction in all nine cross comparisons (Fig. 2, colored dots). This condition was satisfied in 697 of the 3574 probe sets. These 697 probe sets were queried for those that pass the 1.3 fold cutoff in all three cross comparisons of at least one permutation of three cross comparisons (Fig. 2, cyan and red dots). This condition was satisfied by 426 probe sets. These probe sets were ranked by the number of permutations that satisfy the 1.3 fold cutoff. The 1.3 fold cutoff was satisfied for all 84 permutations for 145 probe sets, for 43–83 permutations for 24 probe sets, and for 1–42 permutations in 257 probe sets. The list was cut at the permutation break nearest the expected number of true positives at a 5% FDR (i.e. 180 probe sets). Thus, only the 203 probe sets with ≥35 permutations passing the 1.3 fold cutoff were selected (Fig. 2, red dots). These constitute 6% of all probe sets and corresponded to 8% of known SSTF genes. *Lbx1* repressed 70 and activated 71 SSTF genes (Tables 1, 2). Only 5% are expected to be incorrectly identified interactions. Furthermore, 65 were regulated at least 2 fold and 33 were regulated at least 3 fold. Thus, *Lbx1* causes widespread, and large, perturbations in the transcriptional network of developing dorsal horn neurons in a relatively short time after it begins to be expressed.

**Table 1:** SSTF Genes (1691 genes; 3574 probe sets) Repressed by Lbx1 in the Neural Tube (4%)

Class^a^	#^b^	SSTF^c^	NT d	UCR^e^	Hetero^f^ (n = 3)	Mutant^f^ (n = 3)	Fold Δ^g^							
							(Highest)	Average Signal of PS						(Lowest)
***1. Basic (177 genes; 362 probe sets) 9% repressed***														
bHLH	**1**	**Olig3**	d,vz	137	**56**±*20*	**2207**±*274*	**39.1**							
	2	Bhlhb4	v		**9**±*2*	**129**±*26*	**14.2**							
	**3**	**Neurog2**	d,vz		**931**±*178*	**4346**±*156*	**4.7**							
	**4**	Bhlhb5	dc, vz	33/+	**729**±*50*	**2147**±*245*	**2.9**							
	5	Nhlh1	dc, vz		**1288**±*149*	**2927**±*600*	**2.3**							
	**6**	Npas3	d, vz	125/+	**290**±*18*	**678**±*41*	**2.3**	**2.4**	**2.1**	1.3				
	7	Neurod1	d		**895**±*79*	**1752**±*211*	**2.0**	1.6*						
	**8**	Neurod2	pm	121	**336**±*128*	**497**±*50*	1.5*	1.6*						
	**9**	Neurod4	pm		**351**±*63*	**1541**±*477*	**4.4**	**5.0**	1.2*†					
	10	Tcf15	vz (?)		**140**±*27*	**215**±*13*	**1.5**							
	**11**	Ebf2	pm		**413**±*87*	**617**±*23*	1.1*	1.2*	**1.5**					
	**12**	**Ebf1**	pm	77/+	**1804±** *79*	**2328±** *189*	1.3	1.7*	**1.6**	1.6*				
	**13**	Ptf1a	vz,pm	130	**69**±*41*	**133**±*30*	2.0*							
	**14**	**Ascl1**	d, vz		**491**±*65*	**654**±*58*	1.3	1.6*						
bHLH-ZIP	15	Myc	ns		**101**±*14*	**150**±*3*	**1.5**							
bHSH	**16**	Tcfap2a	dc,pm	30/+	**4175**±*172*	**6205**±*890*	**1.5**	1.5	1.1*					
***2. Zinc-coordinated (749 genes; 1627 probe sets) 2% repressed***														
C4	**17**	Nr4a2	pm	16/+	**526**±*139*	**7015**±*615*	**13.3**	**29.0**	**16.6**	**3.4**				
	**18**	Esrrg	pm	53	**284**±*38*	**1767**±*274*	**6.2**	1.0*†						
C2H2	**19**	Zfp503	+	5/+	**1784**±*236*	**4523**±*698*	**2.5**	**3.0**						
	20	Prdm8	nd	44	**574**±*139*	**1258**±*48*	**2.2**							
	21	Bnc2	nd	25/+	**55**±*17*	**103**±*14*	**1.9**	**1.9**	1.1*†	1.0*†				
	**22**	Sall1	d,vz,pm	+	**711**±*70*	**1324**±*68*	**1.9**	**2.4**						
	**23**	Repin1	ns		**17**±*7*	**30**±*6*	1.2*	1.1*	1.7*	1.2*†				
	24	Zbtb20	d	112/+	**1037**±*49*	**1498**±*164*	**1.4**	1.5*	**1.7**	1.5*	1.4*	**1.6**	1.4	1.7*
	25	BC035954	nd	+	**141**±*25*	**222**±*35*	**1.6**							
	26	Klf7	+	+	**596**±*81*	**958**±*93*	**1.6**	**1.5**	**2.0**	**1.8**				
	27	Zfp703	nd	52	**379±** ***53***	**638±** ***16***	**1.7**	1.0*†						
	28	Zfp319	ns		**279**±*29*	**411**±*49*	1.4	1.2*	**1.5**	1.4*				
	29	Zfp787	ns		**19±** *2*	**27±** *4*	1.3*	1.2*	**1.4†**	1.0*†	1.1*†	1.0*†		
	30	Btbd4	nd		**342**±*26*	**497**±*34*	**1.5**	1.2*	1.1*	1.4*†				
	31	Glis2	vz		**239**±*10*	**326**±*20*	**1.4**	1.1*						
CCHC	32	Peg10	nd		**14**±*6*	**35**±*9*	**2.4†**							
DHHC	**33**	Zdhhc2	ns		**319**±*30*	**565**±*73*	**1.8**	**1.9**	**2.2**	**2.3**				
***3. Helix-turn-helix (512 genes; 1026 probe sets) 7% repressed***														
HD	**34**	Hmx3	dc	29	**9**±*1*	**460**±*96*	**53.3**							
	**35**	Hmx2	dc	29	**10**±*4*	**425**±*20*	**41.2**							
	**36**	**Isl1**	v, dc	+	**233**±*141*	**6128**±*941*	**26.3**	**26.7**						
	**37**	Otp	v, dc	26	**244**±*65*	**6059**±*182*	**24.9**	1.1*†						
	**38**	Phox2b	dc	64/+	**467**±*350*	**4194**±*324*	**9.0**	**3.0**						
	**39**	**Lhx9**	dc	+	**21**±*14*	**54**±*13*	**2.6**	1.1*	1.3*†					
	**40**	Dlx1	ns		**34**±*6*	**53**±*11*	1.6*†							
	41	Pou4f2	d,pm	48/+	**582**±*23*	**3914**±*246*	**6.7**							
	**42**	Pou3f1	dc	59	**339**±*105*	**1728**±*160*	**5.1**	**5.0**						
	**43**	**Pou4f1**	d,pm	79	**5556**±*492*	**10500**±*887*	**1.9**	**2.0**						
	44	Hoxa7	pm	56	**540**±*77*	**921**±*17*	**1.7**							
	45	Hoxc13	v, pm	69	**8**±*1*	**14**±*5*	1.8*†							
	46	Hoxc10	v, pm	69	**284**±*96*	**825**±*64*	**2.9**							
	47	Hoxc9	v, pm	69	**629**±*121*	**1157**±*54*	**1.8**							
	**48**	**Hoxc8**	v, pm	69	**2607**±*346*	**4088**±*305*	**1.6**							
	49	Hoxc6	v, pm	69	**512**±*41*	**850**±*23*	**1.7**	**1.6**	1.3					
	**50**	**Hoxd10**	v, pm	15	**275**±*118*	**618**±*121*	**2.2**							
	**51**	**Hoxd9**	v, pm	15	**492**±*167*	**1157**±*110*	**2.4**							
	52	Hoxd8	+	15	**588**±*147*	**1153**±*226*	**2.0**	**1.8**						
	53	Hoxd1	d (?)	15	**22**±*5*	**41**±*5*	**1.9†**							
	**54**	**Pax3**	d, vz	118	**366**±*77*	**972**±*190*	**2.7**	**2.6**	3.0*					
	**55**	**Pax7**	d, vz	128	**29±** ***2***	**63±14**	**2.2**	1.8*						
	**56**	**Gsh2**	d, vz		**49**±*8*	**104**±*15*	**2.1**							
	**57**	**Gsh1**	d, vz	72	**33**±*7*	**61**±*16*	1.9*							
	**58**	Tgif2	+		**293**±*35*	**391**±*36*	1.3	**1.5**						
	59	Pbx3	pm	7	**8701**±*256*	**12106**±*1173*	1.4	**1.5**						
	**60**	Shox2	pm, dc	42	**143**±7*1*	**634**±45*1*	4.4*	2.4*						
	**61**	**BarHl1**	pm,dc	120	**27**±*1*	**69**±*39*	2.1*	2.6*						
FH	**62**	**Foxd3**	dc,v	17	**15**±*2*	**715**±*33*	**46.9**							
	**63**	Foxp2	vc	9/+	**397**±*121*	**2898**±*273*	**7.3**	**8.3**	**8.0**	**8.5**	**3.9**	1.2*†		
	**64**	Foxp1	m,vc,vz	12/+	**349**±*41*	**1626**±*167*	**4.7**	**5.3**	**4.7**	**4.8**	**5.2**	**4.5**	**4.2**	
	65	Foxp4	pm	128	**77**±*23*	**324**±*77*	**4.2**	**5.2**	1.2*†					
TC	66	Ncor2	v		**250**±*27*	**487**±*100*	**1.9**	**1.8**						
	67	Dll3	vz		**1318**±*198*	**2253**±*36*	**1.7**							
	68	Aste1	nd		**30±** *5*	**42±** *8*	1.4*							
***4. ß-scaffold (116 genes; 281 probe sets) 0.9% repressed***														
HMG	**69**	Sox1	vz		**55**±*5*	**100**±*25*	**1.8**	1.0*†						
***5. Other (138 genes; 279 probe sets) 0.7% repressed***														
	**70**	Dach2	pm	+	**86**±*21*	**196**±*50*	**2.3**	1.8*†						

a Categories and classes according to TRANSFAC (Braunschweig, Germany)

b Bold indicates known nodes or predicted active nodes that behave like known nodes (Kioussi et al., 2006). Underline indicates other predicted active nodes with greater than 1.3 fold partitioning.

c Names according to Mouse Genome Informatics (MGI). Bold indicates SSTFs of the known set (Kioussi et al., 2006).

d Estimated regional expression in the developing neural tube. Estimates are based on expression data linked to the SSTF's MGI website. Expression was observed somewhere between E9.5 and E13.5. Accurate RNA *in situ* data at E12.5 and double-labeling immunohistochemical data are often not available. Codesare as follows: “+”, region specific expression observed; vz, ventricular zone; pm, postmitotic layer or mantle zone; svz, subventricular zone between vz and pm; d, dorsal; v, ventral; dc, dorsalcommissural; da; dorsal association; ns, not seen; nd, no data available.

e Sandelin et al., 2004 lists the 150 largest clusters of ultra conserved regions (non-coding) in the entire mammalian genome (1 is the largest ,150 is the smallest on their list). The number shown indicates the position of this SSTF on their list. “+” indicates that the UCR cluster is enriched in Sox, Pou and homeodomain binding sites (Bailey et al., 2006).

f Values are the average and standard deviation from three microarray values. Data shown is from the probe set with the highest average signal, in all 12 arrays, of those that passed the t-test, or, if none passed t-test, of all.

g Probe sets that passed the 1.3 fold threshold 35 to 84 possible permutations of three pairwise, logical AND comparisons (bold) were selected to establish the initial SSTF gene list. All other probe sets corresponding to these genes were identified in the UCSC genome browser and were added to the table (plain text). Multiple probe sets for a given gene were ranked by their average signal in all 12 arrays an their fold change listed. Asterisk (*) indicates that a probe set failed the t-test at the 95% confidence interval. Dagger (†) indicates that the average signal intensity of the probe set was below 30 (or <0.1% of maximum).

**Table 2:** SSTF Genes (1691 genes; 3574 probe sets) Activated by Lbx1 in the Neural Tube (4.1%)

Class^a^	#^b^	SSTF^c^	NT^d^	UCR^e^	Hetero^f^ (n = 3)	Mutant^f^ (n = 3)	Fold Δ^g^					
							(Highest)	Average Signal of PS				(Lowest)
***1. Basic (177 genes; 362 probe set) 5% activated***												
bZIP	**1**	Mafa	+		**487±** *115*	**48±** *12*	**−10.2**					
	**2**	Mafb	da		**271±** *33*	**64±** *8*	**−4.2**	**−2.6**				
	**3**	Jun	+		**255±** *52*	**129±** *13*	**−2.0**	**−2.1**				
	4	Jundm2	ns	75	**442±** *12*	**313±** *23*	**−1.4**					
bHLH	**5**	**Neurog3**	vz		**25**±*4*	**12**±*5*	**−2.1**	−1.1*†				
	6	Id4	d	+	**7606**±*306*	**3816**±*930*	**−2.0**	**−1.9**	**−1.9**	**−2.5**		
	**7**	Id3	d		**324**±*42*	**191**±*58*	**−1.7**					
	**8**	Hes5	vz		**369**±*62*	**289**±*47*	−1.3*	−1.5*				
***2. Zinc-coordinated (749 genes; 1627 probe sets) 3% activated***												
C4	9	Nr2f2	+	81/+	**833**±*23*	**412**±*66*	**−2.0**	**−2.4**	**−2.0**	**−2.4**		
	**10**	Trps1	d, vz	+	**158**±*32*	**113**±*19*	−1.4*	−1.2*	−1.2*	**−1.8**		
	11	Sall3	da,vz	28	**777**±*184*	**77**±*16*	**−10.1**	**−4.9**				
	**12**	Sall4	+	76	**95**±*28*	**26**±*8*	**−3.7**	−1.5*†				
	**13**	**Zic5**	da	63	**477**±*113*	**63**±*12*	**−7.6**	**−4.5**	**−1.8**			
	**14**	**Zic4**	da	62/+	**414**±*157*	**105**±*18*	**−3.9**	**−3.4**				
	15	Zic3	da	95	**1625**±*212*	**601**±*34*	**−2.7**	**−3.2**				
	**16**	**Zic2**	da	63/+	**327**±*71*	**110**±*7*	**−3.0**					
	**17**	**Zic1**	da	62	**14774**±*804*	**6851**±*417*	**−2.2**	**−3.8**				
	**18**	Wt1	+		**35**±*18*	**10**±*3*	−3.5*†					
	**19**	Zfp804a	nd		**584**±*72*	**195**±*19*	**−3.0**	−1.6*†				
	**20**	Klf5	ns		**33**±*6*	**12**±*2*	**−2.9**	**−1.8†**	−1.5*†			
	21	Zfpm2	pm	21/+	**199**±*54*	**97**±*16*	**−2.1**	−1.4				
	22	Ikzf4	+		**197±** ***12***	**142±** ***16***	−1.4	**−2.2**	−1.1*†			
	**23**	Bcl11a	pm	13/+	**5272**±*514*	**2881**±*141*	**−1.8**	**−1.9**	**−2.1**			
	24	Zcchc11	nd		**1899**±*34*	**1838**±*86*	−1.0*	−1.2*	−1.8*	−1.1*		
	**25**	Hivep2	vz		**153**±*20*	**95**±*5*	**−1.6**	**−1.8**				
	**26**	Rest	da		**187**±*9*	**108**±*13*	**−1.7**	−1.4*	−1.1*†	−1.0*†		
	27	Zfp704	nd		**361**±*52*	**226**±*15*	**−1.6**	−1.2*†				
	28	Zfp467	ns		**227**±*21*	**138**±*9*	**−1.6**	**−1.7**	−1.3*			
	**29**	Zfp775	nd		**208**±*29*	**142**±*11*	**−1.5**					
	30	B930008K04Rik	nd		**62**±*5*	**43**±*6*	**−1.4**					
	31	Zfp784	nd		**54**±*5*	**38**±*2*	**−1.4**					
CCHC	32	Zcchc12	pm		**337**±*71*	**151**±*36*	**−2.2**					
CXXC	**33**	Cxxc5	nd		**3114**±*127*	**1975**±*7*	**−1.6**	**−1.4**				
	34	Cxxc4	nd		**1398**±*118*	**1105**±*129*	−1.3	**−1.4**	1.1*†			
***3. Helix-turn-helix (512 genes; 1026 probe sets) 5% activated***												
HD	**35**	Gbx1	da		**1422±** ***288***	**67±** ***29***	**−21.2**					
	**36**	Gbx2	da	86	**3027**±*237*	**1216**±*169*	**−2.5**					
	**37**	**Lbx1**	da		**3374**±*137*	**331**±*7*	**−10.2**	**−7.6**				
	**38**	Tshz2	d,pm	76	**1372**±*238*	**209**±*24*	**−6.6**	**−8.1**	**−5.3**			
	**39**	**Lhx2**	d,pm	104	**240**±*179*	**34**±*3*	−7.0*					
	**40**	**Lhx1**	pm	50/+	**5763**±*271*	**2575**±*1214*	**−2.2**	**−2.4**				
	**41**	Pknox2	d, pm		**1865**±*198*	**437**±*43*	**−4.3**	**−2.5**				
	**42**	Satb2	+	+	**216**±*28*	**70**±*12*	**−3.1**	−1.0*				
	**43**	**Lmx1b**	da	+	**601±** *135*	**286±** *50*	**−2.1**	−1.1*†				
	**44**	**Nkx6-1**	vz,svz	31	**81**±*27*	**47**±*12*	−1.7*					
	**45**	Hoxa4	pm	56	**149**±*15*	**99**±*4*	−1.7*	**−1.5**				
	**46**	Hoxb2	d,pm	82	**2517**±*160*	**1526**±*130*	**−1.6**					
	**47**	Hoxb8	d,pm	82	**4850**±*249*	**3297**±*476*	**−1.5**					
	**48**	**Msx1**	d,vz		**77**±*14*	**46**±*7*	**−1.7**	1.1*				
	**49**	**Msx2**	d,vz		**14**±*1*	**10**±*2*	**−1.5**					
	50	Zfhx1b	d,svz	2/+	**853**±*100*	**519**±*90*	**−1.6**	**−1.8**	−1.7*	−1.2*†		
	**51**	**Pax6**	vz,da	83/+	**72**±*11*	**51**±*5*	**−1.4**	1.0*	−1.1*			
	**52**	**Prrxl1**	da	101/+								
PD	**53**	Pax8	pm		**861**±*224*	**84**±*24*	**−10.2**					
	**54**	Pax5	+	90	**350**±*45*	**209±** ***24***	**−1.7**	1.2*†	1.0*†			
FH	**55**	E2F8	nd		**53**±*11*	**33**±*7*	**−1.6**	1.0*†				
TC	56	Ets2	d		**346**±*90*	**140**±*22*	**−2.5**					
	**57**	Elk3	+		**103**±*29*	**48**±*14*	**−2.1**	1.1*	−1.3*			
	**58**	Etv5	d		**46**±*5*	**23**±*4*	**−2.0**	1.4*	1.0*†			
WH	**59**	Depdc1a	nd		**31**±*3*	**16**±*3*	**−2.0**					
	**60**	Cdc6	nd		**36**±*9*	**20**±*4*	**−1.8**					
	**61**	Rfx4	d		**524**±*50*	**323**±*86*	**−1.6**	1.1*†				
	62	Myst4	d		**1420**±*39*	**992**±*80*	**−1.4**	−1.1*†				
	63	Rfxdc2	d		**2433**±*54*	**2177**±*121*	−1.1	−1.1*†	−1.6*†	1.0*†		
***4. ß-scaffold (116 genes; 281 probe sets) 2% activated***												
STAT	64	Stat1	ns		**76**±*6*	**53**±*10*	**−1.4**	−1.5*	−1.3*			
HMG	65	Sox13	pm		**127**±*28*	**66**±*7*	**−1.9**					
***5. Other (138 genes; 279 probe sets) 4% activated***												
	**66**	Dmrt3	pm	148	**63**±*31*	**12**±*1*	**−5.5**					
	**67**	Lbxcor1	pm	+	**25659+** *1614*	**6284±** *653*	**−4.1**					
	68	Dmrtb1	nd		**357**±*63*	**169**±*11*	**−2.1**					
	**69**	Notch3	vz		**282**±*82*	**143**±*23*	**−2.0**	−1.3*				
	**70**	Notch2	vz		**266**±*32*	**158**±*33*	**−1.7**	1.0*†				
	**71**	Obfc2a	nd		**186**±*41*	**104**±*15*	**−1.8**	−1.3*	−1.5*	−1.3*†	1.0*†	1.0*†

a–g As in Table 1.

With few exceptions (asterisks), the differences observed in these probe sets were significant at the 95% confidence interval in t-tests. For comparison, 18% of probe sets that showed an average fold change greater than 1.3 and passed the t-test at the 95% confidence interval were not selected by the permutation analysis. In contrast , only 8% of the 203 selected probe sets failed the t-test at the 95% confidence interval (Tables 1, 2; asterisks). Thus, selection by permutation analysis is more stringent than selection by average fold change and t-test. Both methods produce similar lists of targets.

Microarray analysis provided a detailed snapshot of the flux in the transcriptional network when Lbx1 is removed from the system. However, the permutation analysis allows the resolution of the snapshot to be understood intuitively by quantitatively linking the number of selected targets to a FDR. The resolution of the snapshot is limited by the FDR that one considers acceptable. A lower FDR translates to a higher effective cutoff and produces a shorter target list. Nevertheless, demanding excessively low FDRs is counterproductive because it eliminates many true interactions and subtle influences on the network that produce reasonable constraints on an evolving network model. The ability of a given FDR to produce a low cutoff is limited by the ability to reproduce data in biological replicates. For example, the green and cyan dots are closer to the unity line in the white cell comparison (Fig. 2B) than in the green cell comparison (Fig. 2A). These dots represent probe sets whose signals changes in a uniform direction in all single array comparisons, but which fail the 1.3 fold comparison in ≥49 permutations. The genes corresponding to these probe sets were not included in the tables, but potentially have *Lbx1*-dependent expression. One may expect that a larger number of replicate arrays would decrease the cutoff corresponding to a 5% FDR and would allow almost all probe sets to be identified as targets. However, probe sets need to change in a uniform direction in all single array comparisons to be considered in fold scanning analyses. Larger numbers of replicate arrays increase the stringency of this criterion and more probe sets, whose changes are not reproducible or are due to stochastic noise, are thereby eliminated. Permutation analyses allow the quality of different epistasis data sets obtained by microarray analyses to be quantified so that their use in assembling network models can be appropriately weighted.

### Repressed Target Genes

Three target genes, *Foxd3, Isl1,* and *Pou4f1*, are known to be repressed by Lbx1 [9], [30] and appear in Table 1. Foxd3 and Isl1 are normally expressed in postmitotic populations that do not express Lbx1. They are normally not expressed in any of the Lbx1 expressing populations. In *Lbx1* mutants, Foxd3 is specifically upregulated in the dI4L^A^ and I4 populations, and Isl1 is specifically upregulated in the I4L^B^ and I5 populations, respectively The upregulation of each of these genes in only half of the green cells resulted in some of the strongest effects observed. The effects were so strong because none of the green populations initially expressed these genes. Similarly strong effects were observed for the *Hmx2*, *Hmx3*, and *Otp* homeobox genes that have not yet been implicated in the neural tube GRN. The RNA *in situ* patterns of these genes show that their expression is restricted to regions corresponding to the most dorsal postmitotic neural tube populations [32], [33]. Taken together, the results suggest that these three new genes: (1) are expressed in few, if any, of the Lbx1 expressing populations, (2) are strong candidates to play a role in establishing the kernels that specify dorsal cell types, and (3) must be shut down by *Lbx1* in the same way *Foxd3* and *Isl1* are shut down to create the network kernels that specify the dI4-dI6, dI4L^A^ and dI4L^B^ cell types.

The data for the known Lbx1 target gene *Pou4f1* (*Brn3a)* illustrates how striking effects observed in specific populations by immunohistochemistry become moderate effects in microarray analyses because of the pooling of populations. Pou4f1 is normally expressed in dI5 and dI4L^B^, but not in dI4, dI6, or dI4L^A^ [2]. Pou4f1 is specifically upregulated in the dI4 and dI4L^A^ populations of mutants [30]. The early populations each contribute approximately 7% of the green cells whereas the late populations each contribute approximately 40% of green cells. The heterozygous green cells therefore contain 47% cells that express Pou4f1 and 53% that do not. In mutants, only dI6 does not express Brn3a. Thus, 93% of green cells express Pou4f1. The moderate 2-fold increase observed in the microarrays of mutant green populations is consistent with these observations. It becomes clear that the smaller changes observed for many SSTFs may be due to large changes in specific populations that are ameliorated by the pooling of populations in the analysis.

A large number of SSTF genes that have known functions in the establishment of the dorsal progenitor domains show higher expression levels in *Lbx1* mutants (Table 2). These include *Olig3, Neurog2(Ngn2), Ascl1(Mash1), Gsh1, Gsh2, Math1, Pax3, Pax7, and Ptf1a.* These genes are generally expressed in the dorsal ventricular zone and are not co-expressed with Lbx1, which appears shortly after the last cell division [9].

Notably absent from the list of repressed targets are SSTFs that are known to be expressed specifically in nascent postmitotic ventral interneurons (*Evx1, Evx2, En1, Chx10, Sim1, Sox14, Etv1, Etv4, Gata2, Gata3),* motor neurons *(Isl2, Lhx3, Lhx4, Hlxb9 (Hb9, MNR2)),* or the ventral progenitor laminae (*Dbx1, Dbx2, Nkx6.2, Nkx6.1, Irx3, Olig1, Olig2, Nkx2.2, Nkx2.9, Pax6, Gli1, Gli2, Gli3*). Comparisons between mutant and heterozygote embryos with Evx1, En1, and Chx10 antibodies were used to confirm this observation (data not shown).

Taken together, our data suggest that *Lbx1* serves two general roles in downregulating SSTFs in the network. First, it represses SSTF genes expressed in the progenitor pools and thereby destroys network states that confer progenitor cell characteristics. Second, it represses SSTF genes expressed in dorsal commissural interneurons and thereby prevents the establishment of network states that confer properties of relay interneurons. *Lbx1* appears to have little influence on SSTF genes that are expressed in network states that define ventral cell types.

### Activated Target Genes

Five target genes (*Pax2, Lmx1b, Lhx1/5, Satb2, Gbx1*) are known to be activated by *Lbx1* in the developing neural tube [9], [30], [34], [35]. Table 2 shows that *Gbx1*, *Lmx1b*, *Satb2* and *Lhx1* were expressed at significantly lower levels in mutant green cells. The probe set for *Lhx5* produced a robust, but *Lbx1*-independent, signal. There is no probe set for *Pax2* on the array. However, real time PCR analyses showed that *Pax2* RNA declines three fold in mutant green cells (data not shown). Moreover, the two paralogs of *Pax2*, namely *Pax8* and *Pax5*, were expressed at 10.2 and 1.7 fold lower levels in mutant green cells. The present analysis therefore fully confirms and extends the information about known activated targets.

**Figure 4:**
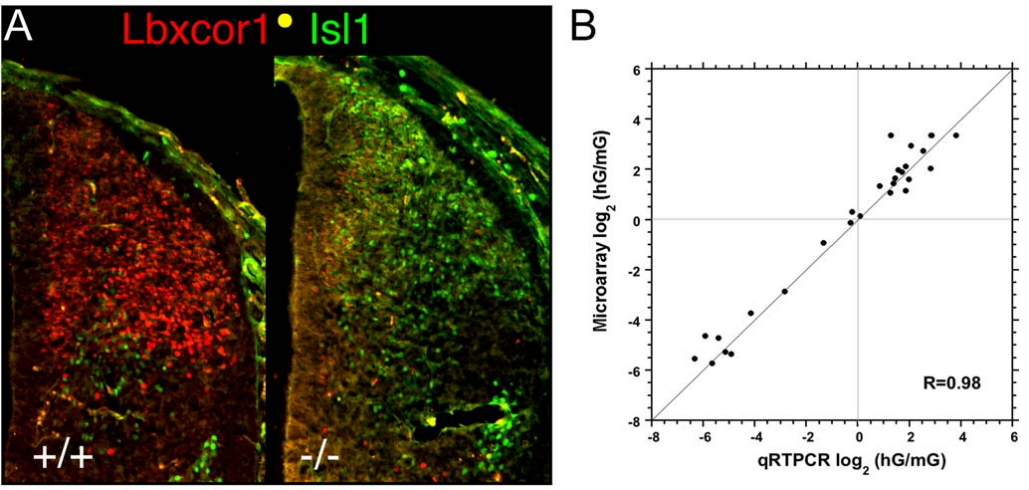
Validation of Microarray Measurements. A) Effect of Lbx1 mutation on Lbxcor1 and Isl1 expression in the thoracic neural tube at E12.5 B) Average fold change observed between hG and mG population pools in three replicate microarrays is plotted against the average fold change measured by quantitative real time PCR (qRTPCR) in at least four replicates. No genes were culled from the initially selected set of 26 genes. Gene regions amplified in qRTPCR generally differed from gene regions detected by Affymetrix probe sets. The outlier in the top right quadrant corresponds to *Mafa*, which gave erroneous low values in qRTPCR because the crossing thresholds occurred after more than 30 cycles. R = 0.99 if this point is disregarded.

The known activated SSTF genes are generally expressed in the nascent dorsal association interneurons that make up the substantia gelatinosa and parts of the nucleus proprius. The expression patterns of *Prxxl1* (*DRG11*), *Lbxcor1(Corl1), Zic1-5*, *Tsh2, Sall3, Zfhx1b*, *Bcl11a* indicate that these SSTFs are expressed in association interneurons [9], [36]–[38]. All of these genes were selected as activated targets (Table 2). Immunohistochemistry was used to confirm that Lbxcor1 was activated by *Lbx1* (Fig. 4A). Thus, *Lbx1* activates SSTF genes expressed in the nascent dorsal association interneurons and thereby promotes the establishment of network states that eventually confer properties of association, or pain-gating, interneurons.

### Lbx1 Represses Ventrally- and Activates Dorsally-Expressed Hox Genes

One striking observation that emerged from the present analysis was the abundance of *Hox* genes and their co-binding cofactors (*Tgif2, Pbx3, and Pknox2*) on the target lists. Three *Hox* genes (*a4, b2*, and *b8*) were activated and ten *Hox* genes (*c6, c8, c9, c10, c13, d1, d8, d9,* and *d10*) were repressed by *Lbx1*. It has long been known that *Hoxb* expression is higher in the dorsal half of the spinal cord [39]. Closer examination of all *Hoxb* probe sets showed that *Hoxb3, b4, and b5* also had significant decreases in expression in the green cells of *Lbx1* mutants. It is also known that *Hoxc6, c8, c9, d9, and d10* gene expression is restricted to postmitotic cells in the ventral region of the neural tube at E12.5 [40]–[43]. The significant increase in the expression levels of these *Hox* genes in *Lbx1* mutants indicates that *Lbx1* represses the expression of these *Hox* genes in at least some dorsal cell populations. The small changes are likely due to the fact that *Lbx1* mediated control of *Hox* genes only occurs in an anterior to posterior (A–P) restricted subset of the green populations for each *Hox* gene. Taken together, the data indicate that Lbx1 plays a key role in controlling which *Hox* genes can function in dorsal cell types and thereby helps to coordinates patterning along the A–P and D–V axes in the neural tube.

### Validation of Target Genes

Quantitative real time PCR was used to validate 25, or approximately 20%, of the identified target set. Primer pairs for *Lbx1*, activated targets (*Pax2, Lmx1b, Pax8, Pax2?e6, Mafa1, Pknox2, Sall3, Tshz2, Lbxcor1, Gbx2, Satb2, Sal4, and Zic1-5*), repressed targets (*Isl1, Foxd3, Olig3, Otp, FoxP2, Hmx2, Hmx3, Nr4a2(Nurr1*), *Sall1*), and neutral genes (*Sall2, Uncx4.1, Bcl11b*) were designed according to the online resource PRIMER BANK. Total RNA from flow sorted green cells from mutant and heterozygote embryos was reverse transcribed and quantitative real time PCR was performed (Fig. 4B). The fold change from four replicate measurements was plotted against the fold change observed for the probe set with the highest absolute signal. The data clearly validate the fold changes observed in the microarray analyses (Tables 1, 2).

### Lbx1 Targets Reside in Clusters of Ultra-Conserved Non-Coding Regions

Perhaps more striking than the large number of *Lbx1*-affected nodes was the observation that 65 of the affected nodes were in the 150 most prominent clusters of ultra-conserved non-coding regions (UCRs) in the mammalian genome [44] and 29 were associated with highly conserved noncoding regions (HCNRs) enriched in Hox, Sox, and POU binding sites [45]. Thus, over half of the *Lbx1*-regulated nodes have previously been associated with genomic regions that are rich in conserved non-coding sequences (Tables 1, 2; UCR column). Many *Lbx1* targets are therefore located in those genomic regions that are richest in evolutionarily conserved regulatory elements, consistent with the idea that Lbx1 participates in the evolutionarily inflexible transcription circuits that are called network kernels.

The clusters of UCRs reported by Sandelin et al. [44]are ranked by density of UCRs within a 500 kb window. The frequency of *Lbx1* targets at the top of the list is higher than at the bottom. Thus, 70% of the first 10, 60% of the first 40, or 50% of all 150 clusters contain SSTF genes with significantly regulated probe sets (t-test; 95% confidence). The SSTF genes located in the genomic regions that are richest in UCRs are likely to have the most *cis-*regulatory modules, and can therefore participate in more, or more diverse, network kernels. Those genes at the top of the list are more likely to have more different roles in development as a whole. This may explain why the frequency of *Lbx1* epistasis rises with UCR density.

## Discussion

The present analysis shows that a single node, *Lbx1*, in a very discrete phase of neural tube development, in a very limited set of neural tube populations, alters expression of 20–30% of the 500–700 active nodes of the transcriptional network of neural tube patterning. Thirty of the 141 SSTF target genes have already been implicated in neural tube development and 85 were predicted active nodes [19]. Only 26 targets were neither known nor predicted nodes. *Lbx1* regulated 46 of the 229 homeodomain and 18 of the 96 basic helix-loop-helix (bHLH) containing genes in the genome (20%). It is known that 82% of the SSTFs used to describe specification in neural tube pattern formation contain either a homeodomain (65%) or a bHLH domain (17%) [19]. The large number of targets and their close association with clusters of UCRs indicates that SSTF nodes may be frequently re-used by different network kernels during development.

The interaction between *Lbx1* and a high fraction of the available SSTF nodes, the strong association of *Lbx1* target SSTFs with the densest UCR clusters, and the ability to observe combinatorial codes by immunohistochemistry are inconsistent with standard hierarchical models of cell lineage specification, in which “master regulators” at the top of the lineage control hierarchies of lineage-specific SSTFs. Instead, they support the view that a central conserved network exists that assumes many different stable states during pattern formation. These stable states are defined by the regulatory interactions between the expressed SSTFs and the kernel-specific *cis*-regulatory modules of these expressed SSTFs. The dense UCR clusters associated with many of the *Lbx1* targets are likely to contain such *cis*-regulatory modules. Collectively, these regulatory interactions stabilize the expression levels of the SSTFs that define the stable network state, which can also be described as a body part, cell type, or network kernel. Transitions between such stable states are likely to be internally clocked or brought on by inductive cues that alter the expression of one or more of the kernel's SSTFs.

During the pattern formation phase of neural tube development, the “combinatorial codes of SSTFs” observed by immunohistochemistry are more consistent with “stable states that rapidly convert to other stable states” than with “smooth or gradual modifications of state” (see below). This paradigm likely reverses at later stages of development when the “stable state” or cell type has been more firmly established.

It is not immediately obvious how a single SSTF perturbation can affect the expression of so many other SSTFs in so few populations in such a short time. One possibility is that rapid signaling cross talk amongst green cell populations alters gene expression levels. However, the lack of cross talk between green and white populations strongly suggests that little signaling cross talk between populations exists at this stage.

A second attractive model to explain the large number of genetic dependencies is that they are not all direct molecular targets. In this model, Lbx1 would regulate the transcriptional output from a small number of the tabulated SSTF genes by direct interactions with their enhancers and these direct SSTFs targets would, in turn, regulate some of the other tabulated SSTFs, as secondary targets. Each level of direct control must alter the protein levels produced from the target gene and must therefore require time for transcription and translation. Approximately 20% of the analyzed cells came from early populations (dI4-dI6) and had therefore expressed Lbx1 for more than 24 hours, which may be sufficient for secondary target regulation. It should be noted that the five fold dilution of RNA from early cells, by RNA from late born cells (dI4L^A^, dI4L^B^), could reduce the ability to detect early *Lbx1* targets.

At least one modeling study suggests that SSTFs do not need to reach steady state levels to alter developmental network state [25], suggesting that 24 hours may be sufficient to allow secondary or higher order effects to be observed. If this were true, then standard immunohistochemistry, which can be done in half-day time scales, would only reveal relatively stable GRN features. Microarray snapshots of epistasis such as those generated by ANCEA, which quantitatively record fold changes, may be the only current way to detect GRN features that change in more rapid timescales.

A final way to explain the large number of SSTF targets is by noting that *Lbx1* regulates different sets of target genes in each population where it is expressed. While it is formally possible that all of the tabulated SSTFs are regulated in all green cells, it is known that Foxd3 and Isl1 are upregulated, and Pax2 and Lmx1b are downregulated, in different populations [9]. It is also formally possible that each cell population uses a discrete non-overlapping set of the target genes. However, Lbx1 regulates Foxd3 and Isl1 in three (dI4, dI6, dI4L^A^) and two (dI5, dI4L^B^) populations, respectively. It is therefore likely that the set of *Lbx1* target genes in each of the five different populations overlaps somewhat with the sets used by each of the other populations. The degree of overlap is likely to be higher in populations that have closer lineage relationships. Thus, it is even possible that some of these target genes function in limb muscle precursors, which also express Lbx1. However, due to the remote lineage relationship a relatively small overlap is expected. Based on these considerations one would predict that Lbx1 regulates between 25 and 141 SSTF genes in each D–V cell type. *Hox* codes could create more cell types by subdividing the D–V cell types along the A–P axis. Thus, even fewer targets per cell type would be predicted.

### Assembly of a Network Model

Constructing and managing a 500–700 node GRN that simulates the emergent properties of neural tube pattern formation requires computer software such as Biotapestry [22] in which expression and epistasis data are integrated into an evolving model as they are acquired. As a first step, one must assemble a “view from the genome”. This requires all nodes and their known epistatic relationships to be entered on one page. Typically nodes that are expressed in related cell types are spatially clustered so that the future “views from the cell” will be spatially coherent.

**Figure 5:**
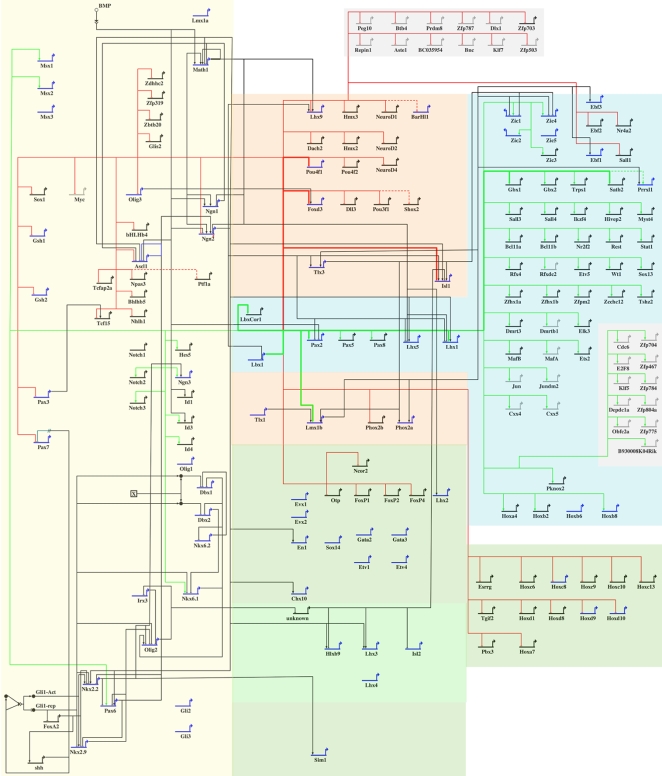
Draft GRN for Neural Tube Patterning. Biotapestry was used to create a *View from the Genome* from known (blue) and *Lbx1*-responsive (black or grey) SSTF nodes and their epistatic interactions. The shaded areas represent different regions of expression: ventricular zone (yellow); ventral postmitotic (green); dorsal commissural (salmon); dorsal association (blue); no expression information (grey). Epistatic relationships between nodes that have been published are shown in black. The epistatic relationships of *Lbx1* that were discovered in this work are shown in green (activating) or red (repressing). Those that were previously known and confirmed in this work are shown as bold red or green lines. We emphasize that the interactions shown are genetic and do not represent direct molecular interactions. Few, if any, direct molecular interactions have been demonstrated in the neural tube literature.

Fig. 5 shows the “view from the genome” of the current network model of neural tube patterning. We have placed all of the known nodes (blue) and new nodes (black) according to their approximate zone of expression (shaded regions). Tables 1 and 2 list many SSTFs whose function in neural tube development has not been explored. Online resources in Mouse Genome Informatics (MGI) and published literature were searched for expression analyses for these target genes in the developing neural tube. Spatially restricted expression patterns in the developing neural tube were found for 57 of the 70 genes in Table 1 and for 54 of the 71 genes in Table 2. Expression patterns from the literature generally were not from the desired cross sections of E12.5 neural tubes. However, crude evaluations of their expression zone were made (column NT in tables). Most of the genes in Table 1 appeared to be expressed outside of the zone of Lbx1 expression, consistent with the idea that they are repressed by *Lbx1*. Similarly, most of the genes in Table 2 appeared to be expressed within the zone of Lbx1 expression, consistent with the idea that they are activated by Lbx1. Those genes for which no expression information could be obtained were grayed out and placed at the periphery of the model.

The background network of Fig. 5 attempts to capture the knowledge base of known nodes and their interactions from the literature. Superimposed in color on this background are the new interactions revealed by this study. The number of new nodes and interactions is far greater than the known ones, demonstrating the efficiency of the ANCEA method. There is also a clear interlocking of the two data sets because 30 of the known nodes are *Lbx1* targets and because virtually all of the known *Lbx1* interactions were recapitulated in the ANCEA dataset. This interlocking provides further validation for the ANCEA method.

The model, it should be emphasized, is only a first crude estimate of the network. The sea urchin endomesoderm models, in which network kernels were discovered, reveal how expression and epistasis information can be coupled in Biotapestry, to give a series of “views from the cell” that show how the network progresses from state to state in time and space [22]. The expression level of each SSTF node is entered for each cell type, at each time in development. The relatively simple and rigid anatomy of the sea urchin embryo allows cell type to be independently identified by anatomical position. In contrast, cell types, or populations, in neural tube development are primarily defined by combinatorial codes of SSTF expression. It follows that a change in the expression status at SSTF node would formally represent the establishment of a new population, cell type, or network state. Given that the set of *Lbx1* targets differs between known populations, it is reasonable to assume that each network state in which Lbx1 is expressed has a different set of *Lbx1* targets. If this is a general property of SSTFs, or only of homeodomain SSTFs, it will be impractical, or perhaps impossible, to construct the network model using only immunohistochemical techniques.

### Coupling ANCEA with PPA to Resolve Networks in Fluid Anatomy

In rigid anatomies, cell populations can be identified by location so that changes in SSTF expression can be tracked over time, or after genetic perturbations. In fluid anatomies such as those of mammals, landmarks that allow populations to be identified only by location generally do not exist during embryonic pattern formation. This problem is particularly acute in the developing central nervous system, where cell populations move and position themselves relative to other populations based on their specifications. Thus, location becomes almost useless as a means to identify populations.

As a result, populations are typically defined by the combination of SSTFs they express. However, the SSTFs they express regulate each other and also define the function of the population. Thus, both the definition and function of a population are inextricably linked to the transcriptional network state of that population. Independent or singular markers of populations do not exist. Mutation of a SSTF to discover its “function” in a population, will generally alter expression of the other SSTF genes used to identify the population. How can one disentangle such a fluid *system* and resolve it into a branching time series of *states* without the aid of spatial landmarks?

Given that the *states* of the *system* are typically defined by combinations of SSTF expression, it seems logical to first determine which SSTFs are differentially expressed within the system. Any SSTFs that are uniformly expressed or silent throughout the system will not contribute to the dynamic description of the states of the system and should be eliminated from consideration. We will call these SSTFs *passive nodes* of the network model of the system. In contrast, SSTFs that are differentially expressed within the system can contribute to the dynamic description of the states of the system and we will call these *active nodes*.

In our previous work [19] we describe a method, called population partitioning analysis (PPA), to systematically identify the active nodes of a system. It requires that one know a SSTF that is differentially expressed within the system. Preferably, the expression pattern of this SSTF should divide the system into two roughly similar sized pools of populations, one pool that expresses the SSTF and one pool that does not. The system must then be experimentally separated into these two pools and the expression of all SSTFs compared between the pools. Passive nodes, being uniformly expressed throughout the system, will show no differential expression on a per cell basis no matter how you divide the system. Active nodes, being expressed in some populations but not in others, will show different levels in the two pools. We demonstrated this by comparing green (Lbx1^+^) and white (Lbx1^−^) population pools in the heterozygous (Lbx1^GFP/+)^ neural tube system [19].These studies indicated that 500–700 SSTFs were differentially expressed in this system and identified approximately 200 of these active nodes.

Once the active nodes of a system have been identified, one needs to know the interactions between the active nodes to set up a dynamic network model of the system. One must bear in mind that *the interactions of an active node may differ in different states (i. e. populations) of the system*.

At the molecular level, interactions between active nodes involve one SSTF protein binding a *cis*-regulatory module that regulates expression of the transcript of a target SSTF. However, dealing with direct interactions at the molecular level is currently intractable. The location of *cis* regulatory modules is generally not known and measuring the effect of SSTF occupancy on them in a particular population (i.e. state) of the system is not currently feasible.

At the genetic level, interactions between active nodes involve mutating the gene for an upstream SSTF and observing changes in the expression of a target SSTF. This establishes that the mutated SSTF is epistatic to the target SSTF. However, the epistatic interaction established in such an experiment is not universally valid throughout the system. It must be linked to a particular state (i.e. population) of the system. If the target SSTF is one of the SSTFs used to define the population (i.e. state) of the system, then one may not be able to link the epistatic interaction to the population because the population (i.e. state) disappears as a result of the technique (mutation) used to measure it. One must have a way of identifying the equivalent population of cells in the presence or absence of the upstream SSTF.

One of the most powerful means to do this is to make a knock-in at the upstream SSTF locus, in which a reporter such as GFP replaces the open reading frame of the SSTF. This allows one to identify equivalent cells in heterozygotes and mutants and therefore allows target gene expression to be compared in these cells in the presence or absence of the SSTF. This is often done by immunohistochemistry or it can be done by the ANCEA method we have developed here. It is critical to do the analysis shortly after the onset of reporter expression so that the measured effects will be primary rather than secondary. In ANCEA one constrains the search for epistatic interactions to the populations (i.e. states) that express the active node one is testing. For example, in this report, we compared gene expression of heterozygote *Lbx1^GFP/+^* green cells with null *Lbx1^GFP/GFP^* green cells to discover epistatic interactions between Lbx1 and target SSTFs. The interactions discovered in this report must occur in one or more of the five populations labeled green by the *Lbx1^GFP^* allele. Thus, the ANCEA method will typically assign the epistatic interactions it discovers to a pool of populations rather than to an individual population (i.e. state).

Ideally, one would like to perform ANCEA on a homogeneous population so that discovered interactions would pertain to only one state and could be input into a “view from the cell” in the network model. Unfortunately, individual populations (i.e. states) are rarely, if ever, defined by the expression of a single SSTF. Instead, individual populations are defined by the combination of SSTFs they express. Most SSTF^GFP^ knock-ins are likely to label several populations (i.e. states) in a local piece of anatomy. One or more additional SSTF markers will generally be needed to identify individual populations.

One future refinement would be to purify cells from mice bearing multiple fluorescent knock-ins of different colors (e.g. SSTF1^GFP^|SSTF2 ^dsRed^). Such an approach would more severely limit the number of populations that interactions could be assigned to in a given ANCEA experiment. The approach would be limited by the fraction of useful embryos in each litter.

A second approach would be to mutate one SSTF while sorting cells labeled by another SSTF. For example, the same Lbx^GFP^ cells could be purified from embryos that are wild type, heterozygote and mutant at another SSTF locus. This approach would be most useful with active nodes that do not interact with the GFP-tagged node.

Applying ANCEA to the active nodes identified by PPA will rapidly identify comprehensive sets of interactions for each active node and assign these interactions to specific pools of populations. Practically this can be done by purifying and analyzing population pools from mutant and heterozygotes of GFP knock-ins at known active nodes, as we have here for *Lbx1*. The results must be integrated with knowledge about combinatorial codes of SSTF expression to assemble network models computationally.

If the large number of genetic dependencies observed for *Lbx1* is typical for all, or just homeodomain containing, SSTFs, then the patterning GRN that generates cell diversity in neural tube is far more cross-linked and complex than was previously appreciated. Either *Lbx1* is a highly connected hub in some or all of the five populations that are currently defined, or there are far more populations in which *Lbx1* participates in only a few of interactions we discovered.

## Materials and Methods

Cell sorting, RNA isolation, probe preparation and quantitative real time PCR (qRTPCR) were described previously [19]. Corl1 antibody was obtained from Yuichi Ono and immunohistochemistry was performed as described [29]. Annotations and permutation analyses were performed by scripts written in the business relational database software FileMaker Pro 6.0. Electronic databases used in this work will be shared.

### Permutation Fold-scanning Analysis

Below we will describe only the analysis of the heterozygous (control) and mutant (test) green samples. The analysis for the white samples was done identically (substitute W for G in the sample names in the text below).

#### Internal- and Cross-Comparisons

In the description below, individual arrays are compared, in a pair-wise manner, within conditions (internal-comparisons; biological replicates) and across conditions (cross-comparisons, test vs. control, between mutant and heterozygote). The six internal-comparisons are hG1 vs hG2, hG1 vs hG3, hG2 vs hG3, mG1 vs mG2, mG1 vs mG3, and mG2 vs mG3. The nine cross-comparisons are hG1 vs mG1, hG1 vs mG2, hG1 vs mG3, hG2 vs mG1, hG2 vs mG2, hG2 vs mG3, hG3 vs mG1, hG3 vs mG2, and hG3 vs mG3.

#### Selecting a Uniformly Changing Set of Probe sets

Only probe sets that changed in a uniform direction (up or down) in all six internal-, or all nine cross-comparisons were considered in the fold-scanning analyses below.

Selection of a set of probe sets that changes uniformly in all six internal comparisons was based on the following type of logic: {IF [(hG1>hG2) AND (hG1>hG3) AND (hG2>hG3) AND (mG1>mG2) AND (mG1>mG3) AND (mG2>mG3)]**OR** [(hG1<hG2) AND (hG1<hG3) AND (hG2<hG3) AND (mG1<mG2) AND (mG1<mG3) AND (mG2<mG3)] THEN “select probe set for further evaluation”}. The three replicate hG arrays can be numbered (1,2,3) in six different ways. Similarly, the three replicate mG arrays can be numbered (1,2,3) in six different ways. As a consequence there are 36 equivalent ways to produce a list of probe sets that change in one uniform direction (only all up or only all down). If one allows two uniform directions (either all up or all down) then half of these 36 ways become redundant. Thus, there are 18 ways to arrange the data and produce a list of probe sets that change in a uniform direction, either all up or all down. For Green internal comparisons, the 18 lists contain an average of 194±109 probe sets. The longest list contains 558 probe sets and the shortest list contains 109 probe sets. {For White internal comparisons, the 18 lists contain an average of 195±74 probe sets The longest list contains 345 probe sets and the shortest list contains 108 probe sets.} Each list produces similar results when subjected to permutation fold scanning analysis (data not shown). However, we conservatively chose the longest list to maximize the measured error. Thus, the uniform list used for generating the internal comparison curves in Fig. 3A and Fig. 3B contained 558 and 345 probe sets (out of the 3574 SSTF probe sets), respectively. These probe sets changed in a uniform direction in all six internal comparisons.

In contrast, there is only one way to select a set of probe sets that change in a uniform direction in all nine cross-comparisons. Selection of probe sets was based on the following logic: {IF [(hG1>mG1) AND (hG1>mG2) AND (hG1>mG3) AND [(hG2>mG1) AND (hG2>mG2) AND (hG2>mG3) AND [(hG3>mG1) AND (hG3>mG2) AND (hG3>mG3)]**OR** [(hG1<mG1) AND (hG1<mG2) AND (hG1<mG3) AND [(hG2<mG1) AND (hG2<mG2) AND (hG2<mG3) AND [(hG3<mG1) AND (hG3<mG2) AND (hG3<mG3)]THEN “select probe set for further evaluation”}. The selected list would be identical regardless of how arrays are assigned to names in this case. There were 697 and 376 uniformly changing SSTF probe sets in the Green (Fig. 3A) and White (Fig. 3B) cross-comparisons , respectively. It should be noted that nine “logical AND” conditions need to be met to make the uniform set in cross comparisons, whereas only six “logical AND” conditions need to be met to make the uniform set in internal comparisons. Thus, the noise measured by the internal comparisons is again, conservatively, overestimated.

#### Permutation Fold-Scanning

Internal-comparisons were fold scanned in the arrangement that produced the largest uniform set (G = 558 probe sets; W = 345 probe sets). As noted above, there are six possible internal-comparisons that can be made for each probe set. These comparisons were labeled A,B,C,D, E, and F. The fold scanner script asks, for a specific combination of three comparisons (three of the letters A through F), within the set of uniformly changing SSTF probe sets, how many probe sets pass the fold cutoff in all three comparisons. The six letters A–F (*n*) can be combined in sets of 3 (*k*) in 20 ways according to the combinatoric formula ^n^C_k_ = *n*!/((*n*-*k*)!*k!). Thus, there are 20 equivalent ways of performing fold scanning on a given uniform changing set. All twenty are performed by the script and the average and standard deviation at each fold cutoff are plotted in Fig. 3. As the scanner approaches the fold cutoff of 1, the curves rise sharply and closely approach the uniform set size on the Y-axis. This is more apparent as fold scanning is done from 1.1 to 1.01 fold cutoffs (data not shown).

Cross-comparisons were fold scanned on the uniform changing set of SSTF probe sets in each case (G = 697 probe sets; W = 376 probe sets). ). As noted above, there are nine possible cross-comparisons that can be made for each probe set. These comparisons were labeled A,B,C,D, E, F, G, H, and I. The fold scanner script asks, for a specific combination of three comparisons (three of the letters A–I), within the set of uniformly changing SSTF probe sets, how many probe sets pass the fold cutoff in all three comparisons. The nine letters A–I (*n*) can be combined in sets of 3 (*k*) in 84 ways according to the combinatoric formula ^n^C_k_ = *n*!/((*n*-*k*)!*k!). Thus, there are 84 equivalent ways of performing fold scanning on a given uniform changing set. All 84 are performed by the script and the average and standard deviation at each fold cutoff are plotted in Fig. 3. As the scanner approaches the fold cutoff of 1, the curves rise sharply and closely approach the uniform set size on the Y-axis. This is more apparent as fold scanning is done from 1.1 to 1.01 fold cutoffs (data not shown).

#### Computing False Discovery Rates as a Function of Fold Cutoff

Internal comparisons should reveal no changes in ideal or perfect replicates. Thus, any differences in our internal comparisons reflect the combined noise, due either to measurement, sample preparation, or to real differences in “identical” biological samples. Two independent measurements of this combined noise, using G internal or W internal comparisons, produced remarkably similar numbers of changes at all fold cutoffs, as would be expected for measurement noise (open boxes in Fig 3A and B). Averages over all 18 Uniform internal sets are even more similar (data not shown).

If there were no true biological differences between mutant and heterozygote samples, then the cross comparisons should reveal the same number of changes at each fold cutoff as the internal comparisons. This is not the case. There are clearly far more changes at all fold cutoffs. The changes observed at each fold cutoff in cross comparisons include real and noise-related changes. Because averages of three specific cross-comparisons were evaluated in both internal and cross comparisons (as opposed to 3 of 6 vs. 3 of 9), the number of probe sets that showed changes at or above a given fold cutoff were evaluated in a statistically equivalent manner in cross and internal comparisons and were therefore directly comparable. The cross comparisons are signal plus noise (real plus false positives) and the internal comparisons are noise only (false positives). The method did not introduce investigator bias or require assumption of a statistical model and is therefore nonparametric.

The FDR (circles) was calculated by dividing the internal comparison values (false positives) by the cross comparison values (real plus false positives) at each fold cutoff. The number of real positives (triangles) was calculated at each fold cutoff by subtracting the internal comparison values from the cross comparison values.

## Supporting Information

Table S1 Tracking of Flow Sorting and RNA Preparation
(0.07 MB DOC)
