# Supplementary material for: How to Build Transcriptional Network Models of Mammalian Pattern Formation
Source: PLoS One. 2008 May 14;3(5):e2179. doi: 10.1371/journal.pone.0002179 (PMC2527684; doi:10.1371/journal.pone.0002179)
Supplement: Table S1 — Tracking of Flow Sorting and RNA Preparation (0.07 MB DOC) [file pone.0002179.s001.doc]

| **Table S1** Tracking of Flow Sorting and RNA Preparation | | | | | | | | | | | | |
| --- | --- | --- | --- | --- | --- | --- | --- | --- | --- | --- | --- | --- |
| Expt | Label | G^a^ | # of  NTs^b^ | Start  Time^c^ | Sort Time^d^ | Total Time^e^ | GFP | % in Pool^f^ | Cells 10^6^ | Pure ^g^  % | RNA^h^  µg | RNA^i^  µg |
| 20-4 | hG1 | **+/-** | 11 | 13:50 | 35 | 62 | **+** | 42 | 2.5 | 95 | 8 | **4.4** |
|  | hW1 |  |  |  |  |  | **-** | 49 | 3.0 | 98 | 12 | **6.4** |
|  | mG1 | **-/-** | 11 | 14:45 | 25 | 50 | **+** | 47 | 2.0 | 95 | 8 | **4.4** |
|  | mW1 |  |  |  |  |  | **-** | 49 | 2.1 | 98 | 9 | **4.8** |
|  |  | **+/-** | 9 | 15:33 | 27 | 52 | **+** | 48 | 2.7 | 96 | 10 | na |
|  |  |  |  |  |  |  | **-** | 47 | 2.5 | 98 | 11 | na |
| 20-5 |  | **+/-** | 10 | 13:18 | 25 | 63 | **+** | 42 | 2.5 | 95 | 8 | 4.9 |
|  | hW2* |  |  |  |  |  | **-** | 48 | 2.8 | 98 | 12 | **7.7** |
|  |  | **-/-** | 10 | 13:55 | 27 | 66 | **+** | 45 | 2.5 | 95 | 9 | 5.0^†^ |
|  |  |  |  |  |  |  | **-** | 47 | 2.7 | 97 | 13 | 6.6 |
|  |  | **+/-** | 13 | 14:33 | 20 | 53 | **+** | 45 | 2.5 | 97 | 10 | na |
|  |  |  |  |  |  |  | **-** | 47 | 2.9 | 97 | 12 | na |
| 20-6 | hG2 | **+/-** | 7 | 13:17 | 30 | 60 | **+** | 43 | 2.5 | 96 | 8 | **5.4** |
|  | hW2* |  |  |  |  |  | **-** | 50 | 3.0 | 97 | 11 | 7.3 |
|  | mG2 | **-/-** | 7 | 13:51 | 27 | 61 | **+** | 39 | 2.5 | 96 | 8 | **5.6** |
|  | mW2 |  |  |  |  |  | **-** | 54 | 3.3 | 98 | 8 | **4.6** |
|  |  | **+/-** | 12 | 14:28 | 25 | 52 | **+** | 41 | 2.4 | 97 | 9 | na |
|  |  |  |  |  |  |  | **-** | 52 | 3.0 | 97 | 12 | na |
| 20-8 | hG3 | **+/-** | 9 | 13:17 | 29 | 65 | **+** | 31 | 2.0 | 98 | nd | **3.1** |
|  | hW3 |  |  |  |  |  | **-** | 64 | 4.5 | 95 | nd | **5.6** |
|  | mG3 | **-/-** | 9 | 13:54 | 34 | 61 | **+** | 34 | 2.1 | 99 | nd | **3.1** |
|  | mW3 |  |  |  |  |  | **-** | 61 | 3.7 | 97 | nd | **4.6** |
|  |  | **+/-** | 9 | 15:10 | 23 | 50 | **+** | 34 | 2.3 | 97 | nd | 3.7 |
|  |  |  |  |  |  |  | **-** | 59 | 4.5 | 99 | nd | 3.3 |
| * hW2 from experiment 6 passed RNA quality control on the bioanalyser but did not label well; reexamination of bioanalyser run shows a diminished 28S RNA band; it was therefore replaced by hW2 from experiment 5  ^†^ this sample passed RNA quality control prior to Turbo Dnase kit but could not be analysed afterward.  ^na^  not applicable; these RNAs were used as pilots for the DNA removal procedure  ^nd^ not determined; these RNAs were not quantitated prior to DNA removal procedure  ^a^ genotype; determined by observing embryos under fluorescent dissection scope; +/- have fluorescent limb muscle precursors , but mutants do not  ^b^ number of neural tubes used in dissociation; neural tubes were removed with few, if any, dorsal root ganglia, neural tubes extended from bottom of the 4^th^ venticle to the end to the hindlimb level;  ^c^ time of day that dissociations was started; embryos were removed from pregnant dams at 10 AM; embryos and neural tubes were iced until dissociation  ^d^ minutes that cells were being collected on ice at the MoFlo fluorescence activated cell sorter (FACS)  ^e^ minutes from adding dissociation buffer to adding lysis buffer  ^f^ percentage of the total cellular events (within forward and side scatter gates and with proper pulse width) that were GFP^+^ or GFP^-^  ^g^ aliquots of sorted samples were rerun to determine the percentage of GFP^+^ or GFP^-^ cells  ^h^ RNA purified by RNeasy kit was quantified by spectroscopy; standard error was 15%; these RNAs showed high molecular weight hump on above the 28S RNA band, which was removed by TurboDNAse treatment  ^i^ RNA remaining after TurboDNase kit cleanup, bioanalyser runs and spectrophotometric quantitation; this was the amount submitted to the core facility for probe preparation; only 2µg were sufficient to create probes without amplification | | | | | | | | | | | | |
